# Retraction: Nanocurcumin Prevents Hypoxia Induced Stress in Primary Human Ventricular Cardiomyocytes by Maintaining Mitochondrial Homeostasis

**DOI:** 10.1371/journal.pone.0319025

**Published:** 2025-02-04

**Authors:** The PLOS One Editors

After publication of this article [1], concerns were raised about results presented in Figs 2–4 and 7–8, specifically:

- In Fig 2A, the bottom right of the N+C Actin panel appears to have similar features to the top left of the H+C Actin panel, and the H+C Actin panel appears different to the H+C Merge panel.
- In Fig 3B in lane 1 of the Ac-H4 panel, there are patterns in the background which appear highly similar.
- Lanes 1–4 of the β-actin panel in Fig 4B appear similar to lanes 1–4 of the β-actin panel in Fig 8A when flipped horizontally and compressed.
- In lanes 1 and 3 in the p53 panel of Fig 4B, there are patterns in the background which appear highly similar.
- In Fig 7A:
  ○ There appears to be a vertical discontinuity between lanes 1 and 2 in the Hypoxia-mito panel.
  ○ The image background patterns in lane 1 in the H+C-mito and H+NC-mito panels appear blurred compared to the adjacent background areas.
- In Fig 8A:
  ○ Across the Hypoxia panel, there are patterns in the background which appear highly similar.
  ○ There appears to be a vertical discontinuity between lanes 3 and 4 in the H+NC panel.
- In Fig 8C:
  ○ Across the Hypoxia panel, there are patterns in the background which appear highly similar.
  ○ There appears to be a vertical discontinuity between lanes 5 and 6 in the H+NC panel.

The authors did not respond to editorial requests for a response and underlying data. In light of the above unresolved concerns that question the integrity and reliability of the reported results and conclusions, the *PLOS One* Editors retract this article.

All authors either did not respond directly or could not be reached.
